# Investigating the frequency of triploid Atlantic salmon in wild Norwegian and Russian populations

**DOI:** 10.1186/s12863-018-0676-x

**Published:** 2018-10-03

**Authors:** Katarina M Jørgensen, Vidar Wennevik, Anne Grete Eide Sørvik, Laila Unneland, Sergey Prusov, Fernando Ayllon, Kevin A Glover

**Affiliations:** 1 Institute of Marine Research, Postboks 1870 Nordnes, N-5817 Bergen, Norway; 2 The Knipovich Polar Research Institute of Marine Fisheries and Oceanography (PINRO), Murmansk, 183038 Russia; 3 Sea lice Research Centre, Department of Biology, University of Bergen, N-5020 Bergen, Norway

**Keywords:** Ploidy, Trisomic, Triploid, Microsatellite, Population, Fish

## Abstract

**Background:**

Fish may display variations in ploidy, including three sets of chromosomes, known as triploidy. A recent study revealed a frequency of ~ 2% spontaneous (i.e., non-intentional) triploidy in domesticated Atlantic salmon produced in Norwegian aquaculture in the period 2007–2014. In contrast, the frequency of triploidy in wild salmon populations has not been studied thus far, and in wild populations of other organisms, it has been very rarely studied. In population genetic data sets, individuals that potentially display chromosome abnormalities, such as triploids with three alleles, are typically excluded on the premise that they may reflect polluted or otherwise compromised samples. Here, we critically re-investigated the microsatellite genetic profile of ~ 6000 wild Atlantic salmon sampled from 80 rivers in Norway and Russia, to investigate the frequency of triploid individuals in wild salmon populations for the first time.

**Results:**

We detected a single triploid salmon, and five individuals displaying three alleles at one of the loci, thus regarded as putatively trisomic. This gave an overall frequency of triploid and putatively trisomic individuals in the data set of 0.017 and 0.083% respectively. The triploid salmon was an adult female, and had spent 2 years in freshwater and 2 years in the sea.

**Conclusions:**

We conclude that the frequency of naturally-occurring triploid Atlantic salmon in wild Norwegian and Russian populations is very low, and many-fold lower than the frequency of spontaneous triploids observed in aquaculture. Our results suggest that aquaculture rearing conditions substantially increase the probability of triploidy to develop, and/or permits greater survival of triploid individuals, in comparison to the wild.

**Electronic supplementary material:**

The online version of this article (10.1186/s12863-018-0676-x) contains supplementary material, which is available to authorized users.

## Background

Polyploidy, i.e., the development of multiple copies of chromosomes within an organism, occurs naturally and is thought to play a role in the evolution of species [1–3]. A triploid organism, resulting from a type of polyploidy, is one that displays three copies of each chromosome. Triploid individuals can occur naturally as a result of meiotic non-disjunction of chromosomes, or in connection with hybridization between species that have different numbers of chromosomes. Triploidy is lethal in mammals [4], while in some other vertebrates, for example birds [5], lizards [6], amphibians and fish [7, 8], triploid individuals may develop and display relatively normal phenotypes. In many species where triploidy is not fatal, it is often associated with sterility or asexual reproduction, although not without exceptions [7].

From the wild, there is a lack of datasets from different species, which makes rigorous testing of how and why polyploidy develops in the natural environment a relatively unsolved challenge [3]. Fish and frogs tend to breed in freshwater, produce large numbers of gametes, have external fertilization and communal breeding, and a type of gametogenesis which enables production of unreduced gametes [7]. These factors may permit polyploidy to develop, especially if environmental variability is present during the breeding season. However, no clear drivers of polyploidy have yet been identifiable from surveys [7]. Unless the variant ploidies produced by any given mechanism give rise to individuals that are fertile and able to meet like-mutated individuals, then the process of speciation though polyploidy is unlikely to succeed [1].

Atlantic salmon (*Salmo salar* L.) is an anadromous salmonid that inhabits temperate rivers on both sides of the North-Atlantic. This species shows highly significant population genetic structuring throughout its native range [9, 10], coupled with extensive genetic-based life-history variation within and among populations [11]. Atlantic salmon, as for all salmonids, underwent a fourth salmonid-specific vertebrate whole-genome duplication ~ 80 million years ago [2, 8], although the species is effectively considered as diploid. Atlantic salmon is the economically most significant aquaculture species globally, with the worldwide annual production exceeding 2 million tonnes since 2012 [12]. The worldwide production of farmed Atlantic salmon in 2016 was over 1800 times the reported nominal catch of Atlantic salmon in the North Atlantic area where Norway and UK (Scotland) produced the majority of the farmed salmon (78% and 12% respectively of 1.5 million tonnes) [13]. However, genetic interactions between domesticated farmed escapees and wild conspecifics represents a major challenge to environmental sustainability [14]. Consequently, significant efforts have been placed into the development of triploid salmon for aquaculture, that are sterile and thus cannot display direct genetic interactions with wild conspecifics. Triploidy in salmon is typically induced via pressure shock treatment administered to eggs post fertilization [15, 16]. As a result of these efforts, considerable work has been conducted to study the biology and welfare of triploid farmed salmon [17–19]. While the biology of triploid salmon is different to that of normal diploid salmon [16], they may nevertheless produce a relatively normal phenotype, and live to adulthood albeit without viable reproduction [17].

Developments in molecular genetic techniques have allowed new angles of investigation into polyploidy [20]. Microsatellite DNA markers, also known as short tandem repeats, have been used extensively over the past several decades to investigate a wide variety of ecological and evolutionary questions, including delineation of population genetic structure and identification of parental contribution [21, 22]. As microsatellites are highly polymorphic, i.e., they display multiple and often tens of different alleles, individual fish often display unique alleles to each other. In turn, this permits the identification of some types chromosome abnormalities, for example triploidy (revealed by up to three distinct alleles per locus). Microsatellites have been used to evaluate ploidy in e.g. plants [23] and frogs [24], and have been validated against flow-cytometry in Atlantic salmon to identify triploid individuals [25].

By screening multiple microsatellite loci in a large number of individuals, we previously demonstrated that ~ 2% of the Norwegian Atlantic salmon aquaculture production in the period 2007–2014 (peaking at ~ 1.2 million tonnes /year), consisted of triploid fish that had occurred spontaneously (i.e., as opposed to a deliberate induction of triploidy via pressure shock) [25]. Furthermore, in the same study, the prevalence of triploidy was as high as 28% in some of the cages sampled on certain farms, demonstrating that this can occasionally occur in high frequencies. Other examples of spontaneous triploidy developing in cultured Atlantic salmon have also been observed in supportive breeding hatcheries in Norway [26] and Estonia [27]. However, the precise reasons for these variations are still largely unknown [25], and whether or not spontaneous triploidy occurs in salmon the wild is at present completely unstudied.

**Fig. 1:**
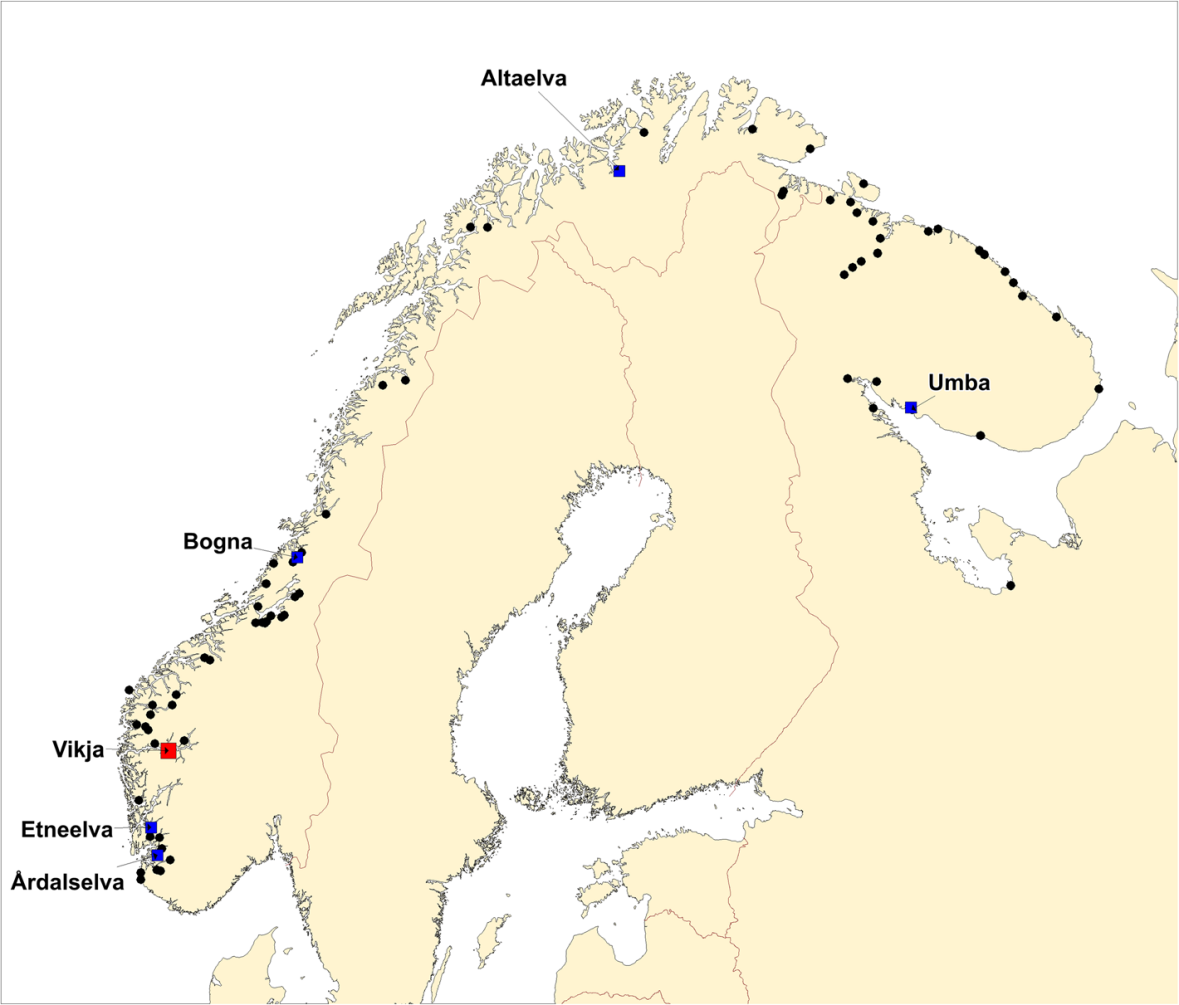
Map of 80 rivers in this study. Rivers are marked with black dots. The river Vikja, where a triploid individual was identified, is marked with a red square. Remaining named rivers, marked with blue squares, were those where trisomic individuals were discovered. The map was based on data from the United States National Imagery and Mapping Agency (NIMA) (http://gis-lab.info/qa/vmap0-eng.html) released into the public domain

Atlantic salmon is a species where a large number of population genetic data sets, often based upon multiple polymorphic microsatellites, exist [28–31]. In such data sets, it is highly common, if not ubiquitous, to exclude individual fish (or genotypes) displaying more than 2 alleles per locus. This is on the premise that the sample is possibly contaminated (i.e., DNA from multiple individuals therefore more than 2 alleles), the third allele represents a technical artefact, or the sample is compromised in other ways. However, close inspection of such samples, combined with re-genotyping for verification, may occasionally reveal that the sample was not contaminated, but was taken from a triploid salmon [25, 32]. Here, we present the results of the first large-scale survey of natural triploidy in a fish population. This was conducted by re-analysing microsatellite genotypes to determine the ploidy of ~ 6000 samples of wild Atlantic salmon collected from 80 rivers in Norway and Russia (Fig. 1).

## Methods

### Samples

The present study is based upon an extensive re-examination of microsatellite genetic profiles of 5994 Atlantic salmon from 56 Norwegian and 24 Russian rivers (Fig. 1, Additional file 1). The samples were collected in two distinct projects designed to study the population genetic structure of salmon in Norway and Russia. The first source of data was from the Kolarctic salmon project datasets of population genetic structure of wild Atlantic salmon in Northern Norway, Northern Finland and Northwestern Russia [33]. In our study, we used samples of exclusively juvenile salmon (0+ − 4+) collected by the electrofishing method in 35 rivers located between 14°E and 60°E (Fig. 1). The second source of data was from an unpublished study of Atlantic salmon population genetic structure throughout Norway and Russia (Wennevik et al., in prep.). In the present study, we used samples from 45 rivers from the unpublished data set (Fig. 1). These samples were taken from a mixture of juvenile and adult salmon (varying ages), collected either by electrofishing in rivers (juveniles) or angling (adults) (Additional file 1). The fish were sacrificed prior to sampling.

### Genotyping and ploidy identification protocol

All microsatellite genotyping was performed at the molecular genetics laboratory the Institute of Marine Research, Norway, and included the analysis of 18 loci [33]. The exact loci, DNA isolation, PCR amplification and electrophoretic conditions to amplify these markers are previously described [33]. This exact set of loci has been used in this laboratory to genotype large numbers of samples over the past decade, including data sets to address population genetic structure [33, 34], investigate pedigree-relationships [26, 34, 35], identification of individual fish [36], and to conduct forensic investigations of farmed escaped salmon back to their farms of origin [37, 38]. These loci have also been genotyped in this laboratory to screen for triploid salmon [25, 32], an approach that has been validated against flow-cytometry identification of triploids [25].

**Fig. 2:**
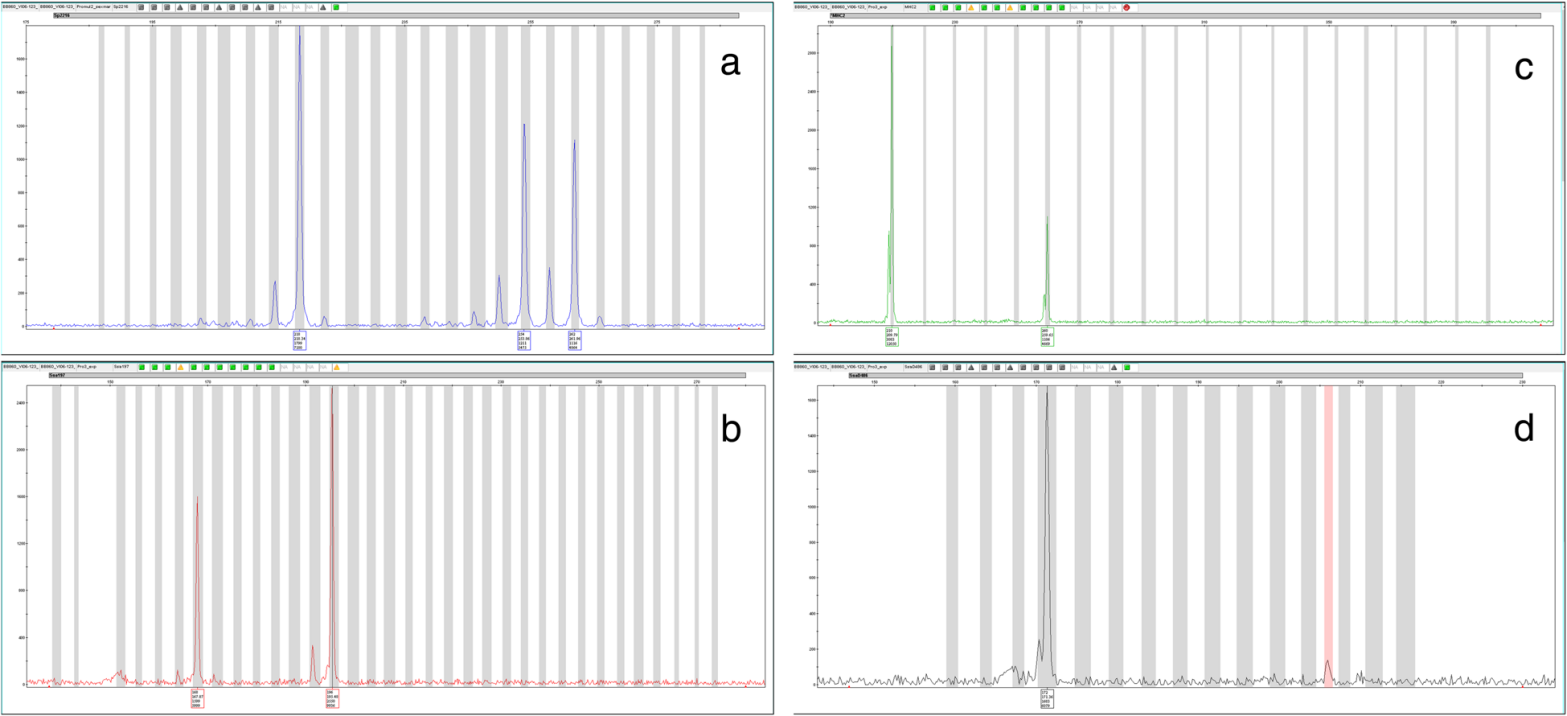
Typical triploid microsatellite marker patterns exemplified by alleles in our triploid salmon individual VI06–123 **a**) Three distinct alleles (Sp2216) **b**) A low (single) allele followed by a high (double) allele (Ssa157) **c**) A high (double) allele followed by a low (single) allele (MHC2) **d**) Three identical alleles (SsaD486)

The microsatellite genetic profiles for the 5994 salmon included in this study were carefully re-analysed in the program Genemapper to identify potential triploids. The implemented protocol for this re-analysis of existing data was very similar to that used to identify triploid salmon in other data sets generated in this laboratory [25, 32]. In short, the protocol involves identification of three distinct alleles at any given locus (Fig. 2a), and/or identification of a higher amplification of the longer allele than the shorter allele (Fig. 2b), which suggests the existence of two copies of the longer allele. From the initial screening of the data set, all individual fish displaying 3 alleles at one or more loci (based upon the above criteria) were chosen for re-genotyping, twice per sample. In cases where results from the three independent genetic analyses of the same sample gave an identical result, the result was considered correct and was permitted to stand. An individual was reported as putatively trisomic if it displayed three alleles at just one of the loci, and triploid if they displayed 3 alleles at 2 or more of the loci analysed. Individuals scored for less than 5 of the 18 markers investigated in the initial data sets were removed from the analysis. Samples containing four or more alleles were classified as contaminated and removed from the data set. It is theoretically possible that some individuals displaying four alleles at a given locus could reflect a form of aneuploidy, i.e., tetrasomy or even tetraploid, as opposed to a polluted sample as classified here. However, this was not investigated in the present study, and we draw therefore no assumptions about this remote possibility.

### Excluding potential species hybrids

The remote possibility that any of the triploid or putative trisomic salmon identified here represented spurious results caused through hybridization between salmon and wild brown trout (*Salmon trutta* L.), which is known to occur in the wild, was excluded. This was achieved by two methods. First, a number of brown trout and trout salmon hybrids have been inadvertently genotyped in this laboratory, and excluded based upon their microsatellite DNA profiles. Especially loci SsaD486 and SSp3016 display non-overlapping alleles between these two species, and thus such individuals are easily identified. However, in order to fully exclude this possibility, all of the triploid and putatively trisomic salmon identified here were genotyped at the diagnostic 5S rDNA locus to for identification of hybrids between these species [39]. Included in this test were reference brown trout and 2 reference Atlantic salmon. Based on their microsatellite profiles and/or phenotypes, 8 brown trout and 4 hybrids from our own material were also included.

## Results

After the extensive re-analysis of the existing microsatellite data from 5994 salmon, one putatively triploid and 12 putatively trisomic salmon were identified (Table 1, Fig. 1, and for full survey results see Additional file 1). All 13 of these samples were re-genotyped, twice, in order to investigate whether the genotype observed in the initial analysis was identical to the genotype in the second and third analysis. This additional analysis confirmed the genotype of the triploid individual, and confirmed that 5 of the 12 putatively trisomic fish had indeed three alleles at one of the loci.

**Table 1:** Main survey results: individuals identified with triploidy or trisomy

River	Sample	Board	Markers																		Comment	Re-run	Evaluation
			1	2	3	4	5	6	7	8	9	10	11	12	13	14	15	16	17	18			
Umba	UMB09–27	BB830														x						Confirmed	Trisomic
Kitsa	KTS09F-9	BB834					x															N.C.	Diploid
Alta	AL05–6-8	BB502						x													Low quality sample	Confirmed	Trisomic
Målselv	ME071–5	BB589								x											Low quality sample	N.C.	Diploid
Bogna	BO07-St1–30	BB661	x																			Confirmed	Trisomic
Vigda	VG07–79	BB570						x													Uncertain	N.C	Diploid
Vikja	VI06–123	BB860				x	x	x		x		x				x		x			Triploid	Confirmed	Triploid
Gjengedalsv.	GV07–35	BB667			x																Low-high	N.C.	Diploid
Gjengedalsv.	GV07–48	BB667			x																Low-high	N.C.	Diploid
Etneelva	ET06–9	BB358			x																Low-high	Confirmed	Poss. Trisomic
Etneelva	ET06–68	BB358						x													Low-high	N.C.	Diploid
Årdalselva	AD09-St1–23	BB766															x				Low-high	Confirmed	Poss. Trisomic
Årdalselva	AD09-St1–30	BB766	x																		Low-high	N.C	Diploid

Markers: 1 = SSsp2201, 2 = SSsp2210, 3 = SSspG7, 4 = Ssa202, 5 = SsaD144, 6 = SsaD157, 7 = Sp1605, 8 = Sp2216, 9 = Ssa14, 10 = Ssa171, 11 = Ssa289, 12 = MHC1, 13 = MHC2, 14 = SSsp3016, 15 = SsOsl85, 16 = Ssa197, 17 = SsaD486, 18 = SsaF43

The remaining 7 putatively trisomic fish were therefore discarded as genotyping errors, inconsistencies, technical artifacts or otherwise inconclusive. The remote possibility that any of the 13 fish re-analysed, were hybrids between brown trout and salmon, was conclusively excluded using the two approaches described in the methods, including the use of the 5S rDNA species-specific diagnostic marker [39]. Therefore, given that 5994 samples were investigated, our results thus reflect an incidence of triploidy in wild salmon populations in Norway and Russia as 0.017%, and an incidence of putative trisomy as 0.083%.

For the individual “Vi06–123” that was confirmed as triploid, eight of the 18 loci genotyped displayed three alleles (Table 1). This was either detected by the presence of three distinct fragments for the loci Ssa202, SsaD157, Sp2216 (Fig. 2a), Ssa171 and Ssp3016, or by the longer allele amplifying greater than the shorter allele for the loci SsaD144, Ssa197 (Fig. 2b), SsOsl85. While the latter does not unequivocally demonstrate three copies, in earlier tests in this laboratory to validate microsatellite genotyping against flow-cytometry [25], such allelic patterns were also clearly associated with triploidy. This triploid salmon was sampled from the river Vikja in county Sogn og Fjordane (Fig. 1), was female, and was confirmed to be a wild individual through scale analysis [40]. Scale analysis further revealed that she spent 2 years in the river before smoltification, and then 2 years at sea before returning to the river where she was captured by rod and line. Unfortunately, the fish was not dissected so it was not possible to look for gonad development and verify phenotypic sex. It displayed an estimated smolt length of 14.8 cm, and an adult length and weight upon capture in the river of 71 cm and 2.3 kg. This is smaller than typically observed for wild salmon that have been in the sea for 2 years.

For the five putatively trisomic individuals (UMB09–27, AL05–6-8, BO07-St1–30, ET06–9 and AD09-St1–23) that had their genotypes confirmed in all three analyses, one out of the 18 microsatellites displayed three alleles. This was either revealed as three distinct alleles for individuals UMB09–27, AL05–6-8 and BO07-St1–30 at the loci SSsp3016, SsaD157 or SSsp2201 respectively, or alternatively, by the longer allele amplifying greater than the shorter allele for individuals ET06–9 and AD09-St1–23, at the loci SSspG7 and SsOSL85 respectively (Table 1). Thus, putative trisomy was associated with different markers for all five cases. There is also a good geographical spread of the rivers they originated from – Alta (AL, Norway, 69,9°N), Umba (UMB, Russia, 66,67°N), Bogna (BO, Norway, 64,39°N), Etne (ET, Norway, 59,67°N) and Årdalselva (AD, Norway, 59,14°N) (Fig. 1).

## Discussion

This study represents the first investigation into the frequency of naturally occurring triploidy in wild Atlantic salmon populations. By systematically re-examining the microsatellite DNA profiles of ~ 6000 salmon collected in 80 Russian and Norwegian rivers, we were able to identify a single triploid salmon (i.e., a fish displaying three alleles at eight of the 18 loci analysed), and five individuals that were classified as putatively trisomic (i.e., fish that displayed three alleles at one of the 18 markers investigated). The frequency of natural triploidy observed here (1 in 5994: 0.017%) was thus observed to be ~ 10 times lower than in escaped farmed salmon recaptured in Norwegian rivers in the period 2007–2014 [32], and ~ 100 times lower than the frequency of spontaneous triploidy observed in domesticated salmon reared on commercial fish farms in Norway in the period 2007–2014 [25]. We suggest that there are two primary explanations for the very low incidence observed in juvenile and adult salmon in the wild, and the large contrast compared to the situation for salmon in aquaculture: 1) spontaneous triploidy is very rare in natural salmon populations, and 2) individuals arising from spontaneous triploidy in the wild display reduced survival rates. These are discussed below.

It has been suggested that in commercial aquaculture or supportive breeding hatcheries, spontaneous triploidy may arise due to over-aging of the eggs prior to fertilization, possibly in combination with increased temperatures of the eggs [8, 41], and mechanical disturbance. These factors are believed to cause increased rates of meiotic disjunction [15]. Delayed fertilization frequently occurs in the fish farming industry due to logistics and management practices [25]. However, in the wild, females will breed “when they are ready”, by releasing their eggs into gravel depressions known as redds, while one or several males simultaneously fertilize [42]. This process means that in the wild, the chances of over-maturation of eggs, either pre- or post- fertilization, are remote, and, the eggs are fertilized in the same water temperature as the water that surrounds the female. Consequently, some of the reasons suggested for the relatively high frequency of spontaneous triploidy occurring in farmed salmon are unlikely to have the opportunity to cause this in the wild.

Cold shock can be used to deliberately induce triploidy in fish farming [15], and has been suggested to also be of relevance in naturally occurring meiotic non-disjunction in connection with rapidly changing weather [7]. Conceivably, the latitude and local climate could then influence triploidy rates in natural populations. However, since we only detected one triploid salmon in the present study, our findings do not allow any conclusions regarding this theory.

Rates of natural triploidy vary greatly among species, however, the reasons for this are unknown. In humans, where triploidy is lethal, the rate, determined from genotyping of spontaneous abortions, is less than 1% [43]. In amphibians, natural triploidy rates between 0.2–16.7% have been reported from a number of smaller studies [44]. However, several species of lizards and salamanders reproduce asexually [6], and some frogs (e.g. *Pelophylax* sp.) have triploid subpopulations that reproduce by mechanisms that are not known in fish [24]. Many other fish species (e.g. *Cypriniformes*, *Gymnotiformes*, *Siluriformes*, *Characiformes*) that are known to have variable ploidy populations also utilize multiple modes of both sexual and asexual reproduction [7, 45, 46]. In contrast, female triploid Atlantic salmon are invariably sterile, while triploid males can produce aneuploid sperm [15]. It is thus highly unlikely that naturally occurring triploid salmon can reproduce in the wild. This may be a factor in explaining the rare natural occurrences of triploidy in Atlantic salmon.

Little direct evidence is available regarding survival rates of triploid salmonids in the wild. However, triploid salmon are known to display reduced swimming endurance, increased temperature sensitivity, poorer disease resistance and impaired stress responses in comparison with diploid salmon [15, 47]. It has therefore been suggested that triploid fish, escaping from fish farms, are to be expected to display higher mortality than diploid escapees [15]. Two studies using different methods of investigation also suggest lower freshwater return rates for triploid Atlantic salmon after escape from farms or deliberate release as smolts [32, 48]. In rainbow trout, lake survival is lower in triploid compared to diploid individuals [49]. Incidentally, the single identified wild salmon in the present study was an adult which has returned from the sea, despite the fact that most of the other salmon investigated were juveniles. If the survival of spontaneously occurring triploid salmon in the wild is lower than for normal diploid salmon, then one would expect that the probability of observing a triploid salmon would decrease with age. This is especially the case as triploid fish display reduced return to freshwater after seaward migration, presumably due to the lack of a maturation signal [32, 48]. In order to investigate whether triploids exist in the wild, but are rapidly selected out of the population, sampling fertilized eggs in redds would represent the ideal survey method. Such a sampling regime could be implemented in follow-up studies to the present.

This study used microsatellite DNA analysis for identifying triploid individuals, a method that has been tested before by us against flow-cytometry [25, 32], as well as by others [23, 24]. Depending upon the parental genotypes, the genotype of the triploid individual for any given locus may manifest itself as: 1) three separate alleles 2) one copy of one allele and two copies of a second allele 3) three copies of the same allele. 1) is readily identified from genotypic data (Fig. 2a), and 2) where there is one copy of the shorter allele and two copies of the longer allele (Fig. 2b), the three copies are readily spotted, although this requires careful inspection of the microsatellite profile and good control over the technical robustness of the markers used to rule out potential genotyping artifacts. The other possibility under 2: two copies of the shorter allele and one copy of the longer allele (Fig. 2c) is very difficult to spot as it may resemble the patterns typically seen for a normal diploid individual. This was not used to determine three alleles in the present study. Finally, using standard microsatellite genotyping platforms, there is no way to identify three copies of the same allele (Fig. 2d). Nevertheless, despite the above challenges to use microsatellites to identify triploid individuals, triploids should have three detectable copies of alleles at many of the 18 loci tested (so long as they are polymorphic loci and it is being investigated in populations or strains displaying genetic variation), meaning that the likelihood of detecting several alleles at some of the loci is very high. Furthermore, since the present study also identified five putatively trisomic individuals, the risk of missing triploid fish is highly unlikely (a simple estimate of the probability of a false negative is 0.5^18^ = 3.8e-6). We therefore conclude that our triploid identification method has accurately estimated the number of triploid fish in the samples used here.

Trisomy is also caused by a failure of chromosome separation, but affecting only a single chromosome [4]. Trisomy is a less severe chromosomal defect and is usually more common than triploidy. Natural aneuploidy (trisomy or monosomy) rates vary greatly between species – curiously, the rates in humans (10–30% of eggs) and mice (1–2%) are much higher than in fruit flies (1 in 6000 eggs) or baker’s yeast (1 in 10.000) despite the severe disabilities associated with trisomy in mammals [4]. Trisomy [4] is at least 10 times more common than triploidy [43] in humans. In mice, the rate of trisomy (aneuploidy) is about 2.5 times that of triploidy in normal IVF eggs – however, this situation is not 100% natural [50]. Due to the manual detection method implemented here, the rate of trisomy, referred to as putative trisomy in our data set, is likely to be underestimated, possibly by as much as 50%, since only 2 out of 4 possible ways of expressing three alleles can be detected with confidence (see discussion above). Also, the microsatellite markers used here do not represent all chromosomes, leading to a further underestimate of trisomy in individuals. The frequency of triploidy and putative trisomy discovered here (1 in ~ 6000 and 5 in ~ 6000) is comparable to the rate of trisomy reported for fruit flies [4]. An approximately fivefold difference in incidence of triploidy and trisomy is also within the range of reported values. Assuming a 50% underestimation of trisomy, as discussed, suggests the real difference could be as high as tenfold – this is still within what is known from other species. No theories currently exist to explain differences in natural rates of the different types of chromosomal non-disjunctions between species.

## Conclusions

In conclusion, we have investigated for the first time, the frequency of triploidy and putative trisomy in wild salmon populations. The observed very low frequency of triploidy revealed in the wild here, demonstrates that the rates of up to 28% seen in some fish farms, is highly likely to be due to the specific conditions of the breeding in that environment, although a role of additional mortality of triploids in the wild cannot be ruled out. Further investigation in the wild, for fertilized eggs could reveal further insights into this.

## Additional files

Additional file 1: Summary of salmon ploidy per river. (XLSX 21 kb)

Additional file 2: Microsatellite genotyping results for all salmon identified as possibly triploid or trisomic. (PDF 874 kb)

## Abbreviations

AD: Årdalselva
AL: Alta
BO: Bogna
ET: Etneelva
UMB: Umba
Vi: Vikja
