# Supplementary figures and images for: Investigating the frequency of triploid Atlantic salmon in wild Norwegian and Russian populations
Source: BMC Genet. 2018 Oct 3;19:90. doi: 10.1186/s12863-018-0676-x (PMC6171226; doi:10.1186/s12863-018-0676-x)

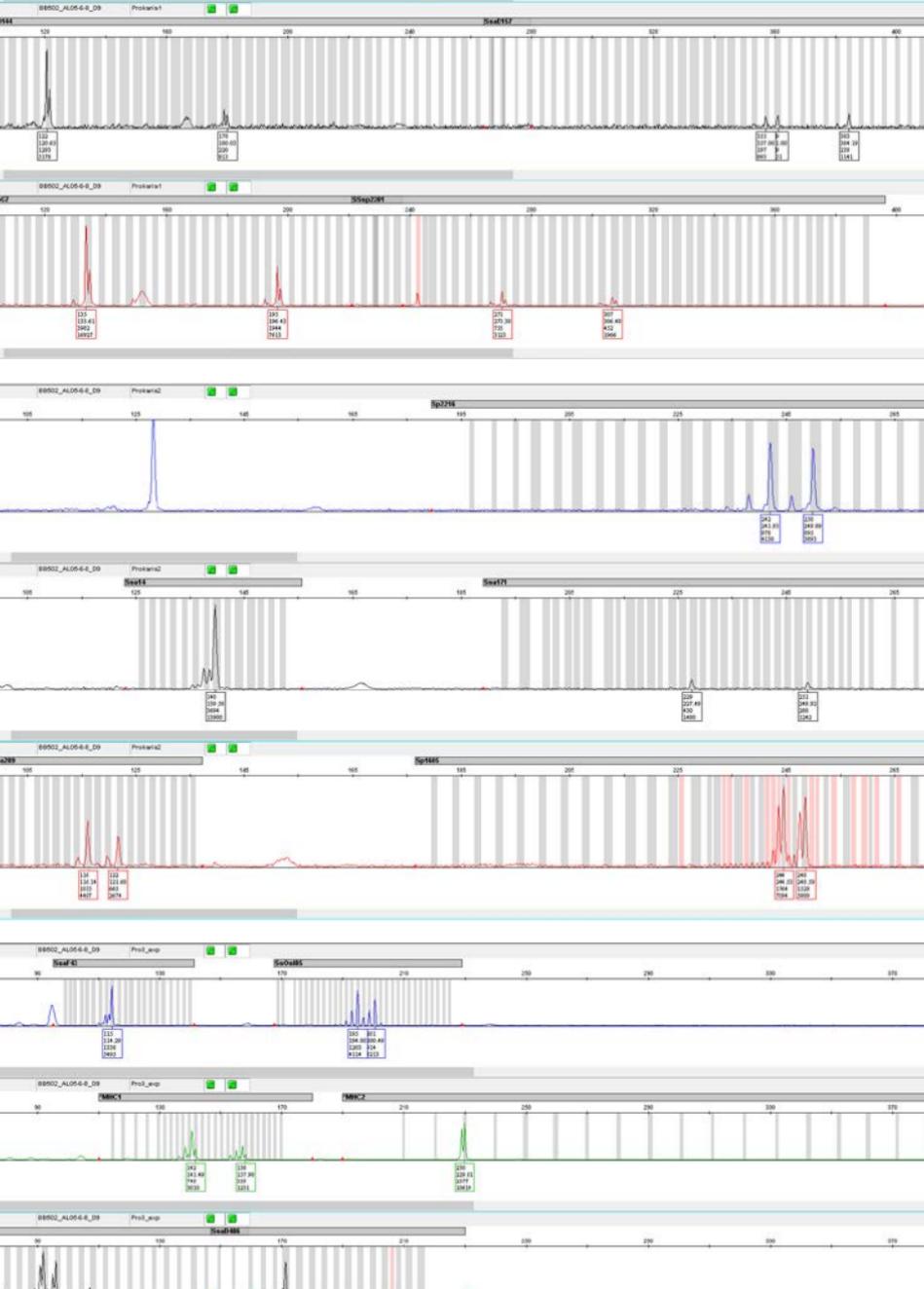

# Bogna\_BB661\_BO07-St1-30

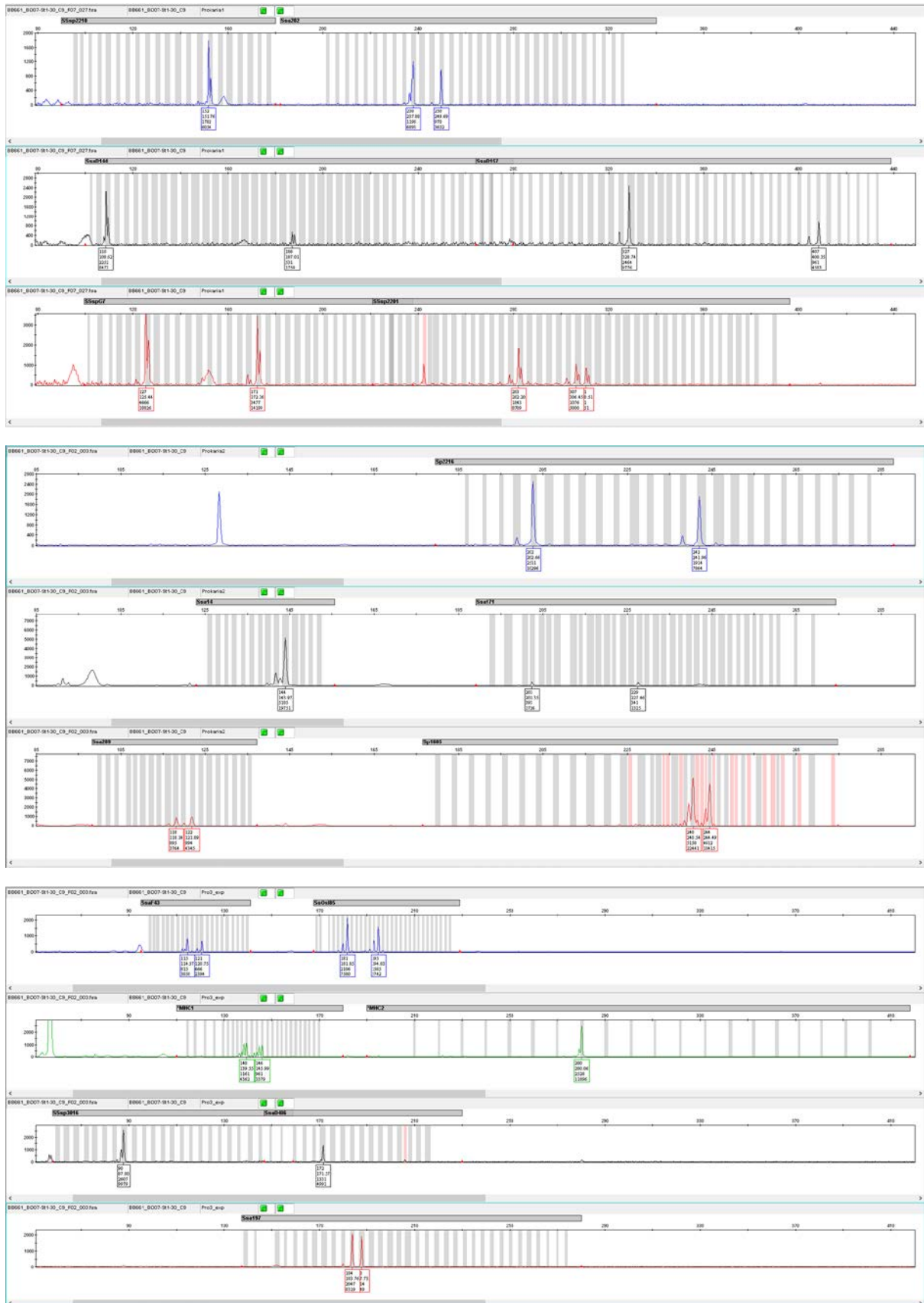

# Vikja\_BB860\_VI06-123

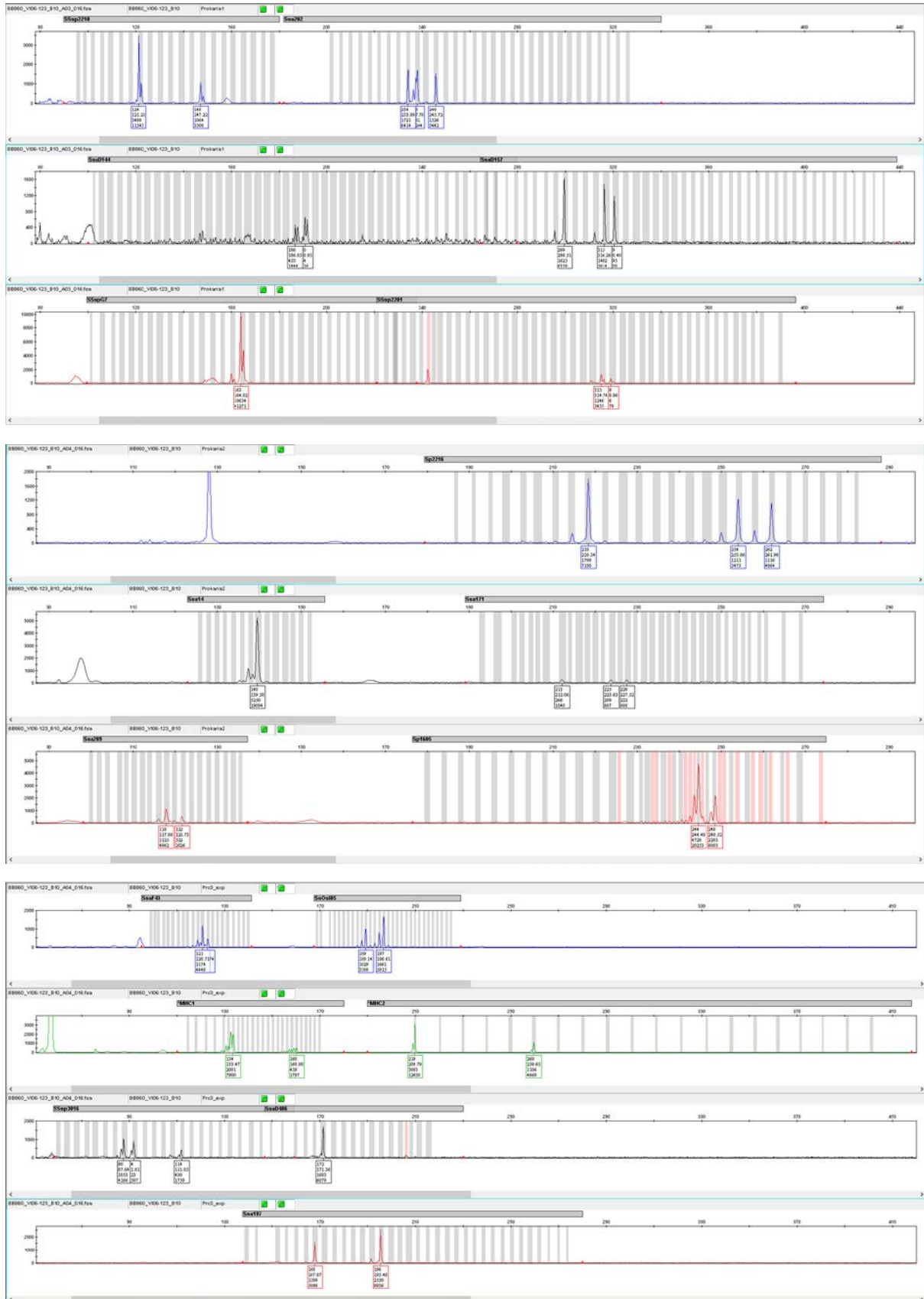

# Etneelva\_BB358\_ET06-9

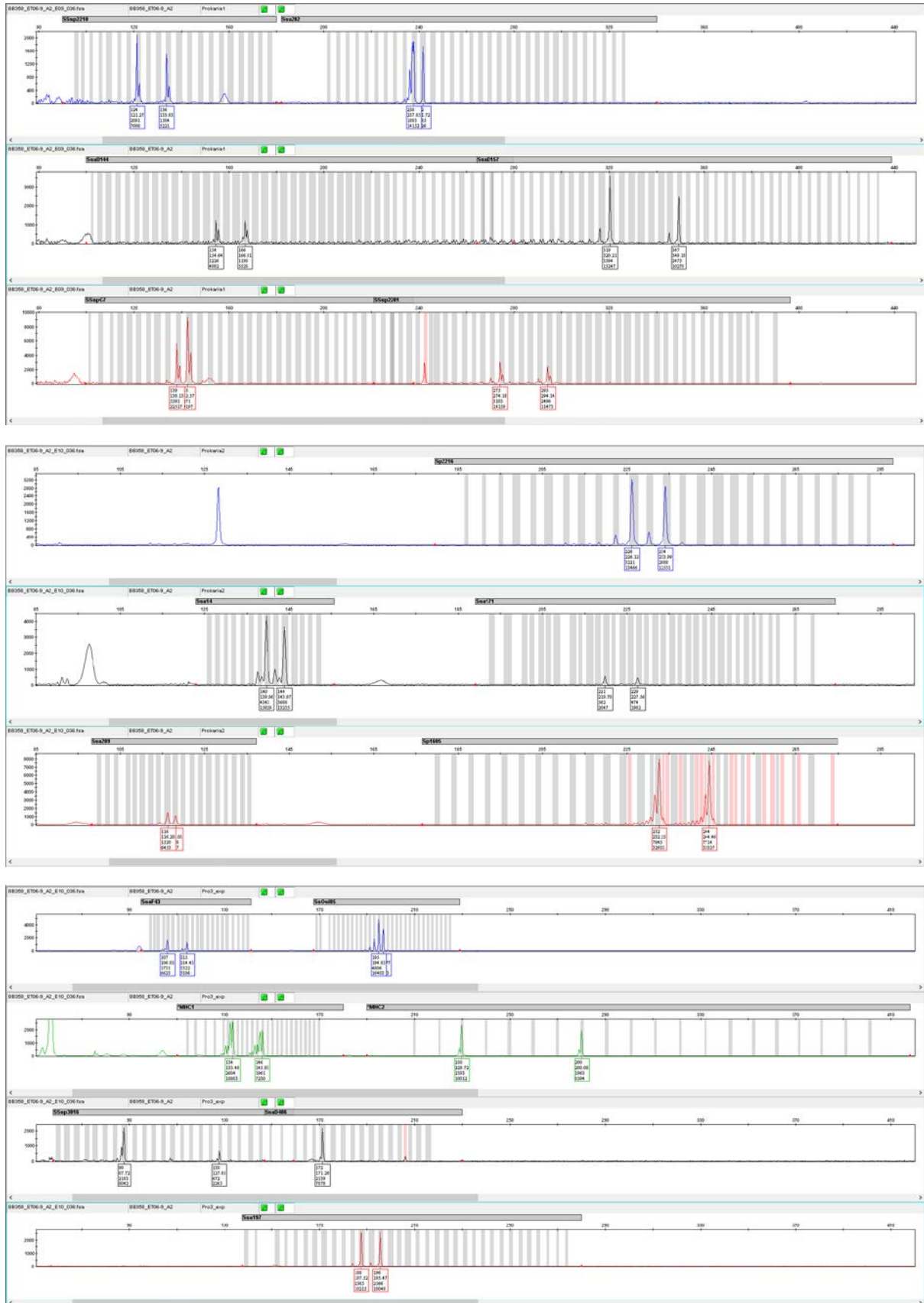

# Årdalselva\_BB766\_AD09-St1-23

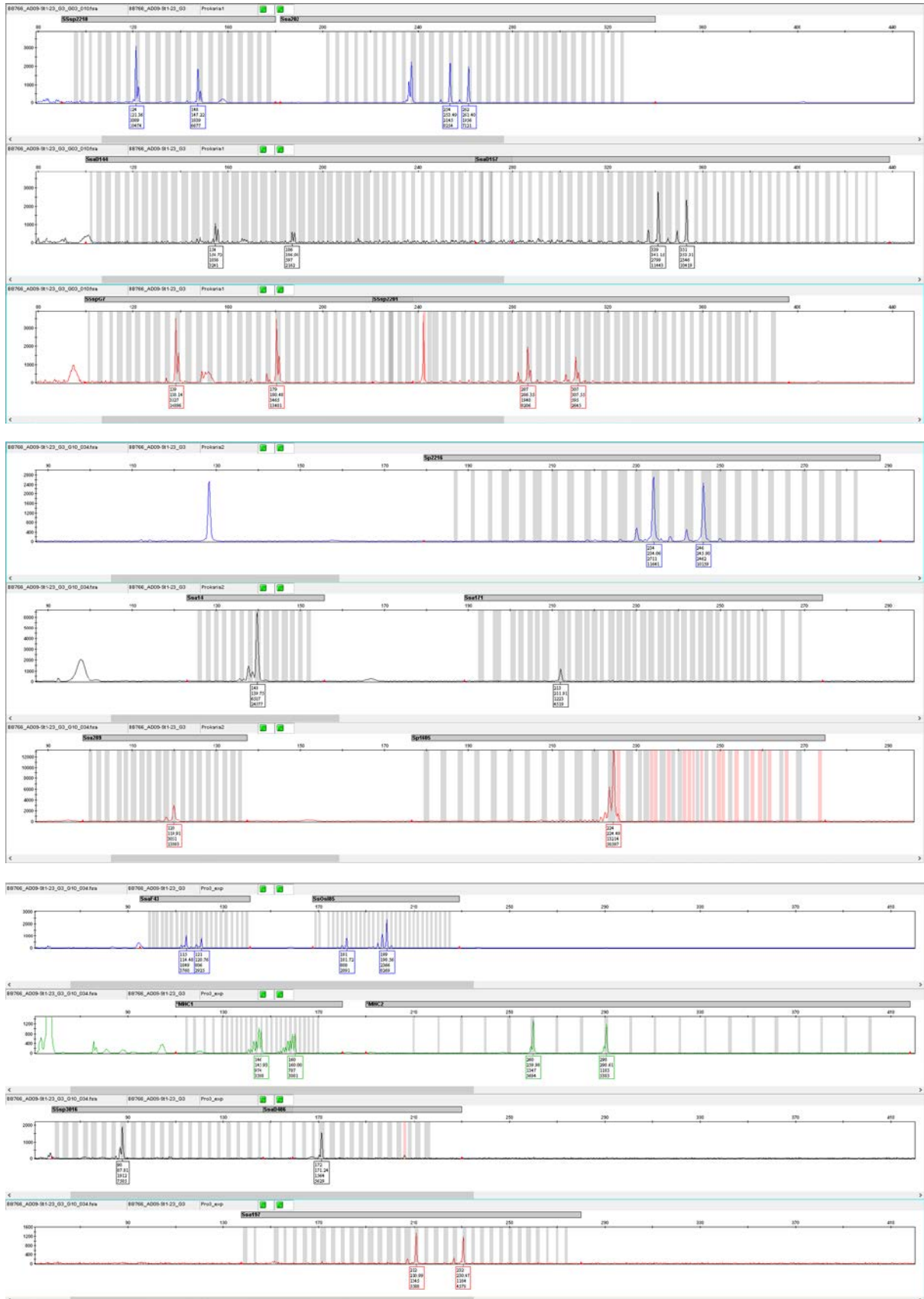

Supplement: Supplementary file 2 — Microsatellite genotyping results for all salmon identified as possibly triploid or trisomic. (PDF 874 kb) [file 12863_2018_676_MOESM2_ESM.pdf]
